# Nurses’ experiences during the COVID-19 pandemic in Iran: a qualitative study

**DOI:** 10.1186/s12912-021-00722-z

**Published:** 2021-10-14

**Authors:** Vahid Zamanzadeh, Leila Valizadeh, Mohammad Khajehgoodari, Farzaneh Bagheriyeh

**Affiliations:** 1 Department of Medical Surgical Nursing, School of Nursing and Midwifery, Tabriz University of Medical Sciences, Tabriz, Iran; 2 Department of Pediatric Nursing, School of Nursing and Midwifery, Tabriz University of Medical Sciences, Tabriz, Iran

**Keywords:** Nurses’ experiences, COVID-19, Crisis

## Abstract

**Background:**

Nurses are at the forefront of patient care during infectious disease pandemics and they play a key role in treating and preventing the upward trend of the disease. Hence, it is crucial to consider their experiences in designing action plans to combat coronavirus disease 2019 (COVID-19). Since there is not enough data in this regard, the current study aimed to investigate the nurses’ experiences in caring for patients with COVID-19 in Iran.

**Methods:**

In this descriptive qualitative study, a total of 20 nurses were selected by purposive sampling. Semi-structured interviews were conducted and analyzed using qualitative content analysis to collect data.

**Results:**

Data analysis revealed four main themes, including ‘duality in the form of care,’ ‘confusion and ambiguity in care planning’, ‘workload’, and ‘social isolation in spite of positive image.’

**Conclusion:**

Our findings indicated that the nurses experienced a range of paradoxes during the COVID-19 pandemic; these paradoxes included distraction from providing care due to focus on marginal factors in spite of empathy and cooperation in nurses, the presence of volunteer support staff despite the lack of equipment, lack of scientific information and the unreliability of online information, overload in the hospital due to insufficient facilities and equipment, and the physical avoidance of people in the community in spite of social support for nurses in the media. The results of this study can lead to a clear understanding for managers and healthcare policymakers in the country and aid them in taking optimal measures to support nurses and improve the quality of nursing care against COVID-19.

## Background

Coronavirus disease 2019 (COVID-19) was first reported in December 2019 in people with lung infections in Wuhan, China [1]. On March 11, 2020, the World Health Organization (WHO) declared it a pandemic [2]. According to the WHO’s official figures on March 8, 2021, more than 113 million people have been infected with the disease worldwide, and the death toll exceeds 2.5 million. Iran’s share of these statistics is more than 1.8 million infections and more than 62,000 deaths [3].

The widespread outbreak of COVID-19 has put all healthcare systems under pressure, creating unprecedented demand for healthcare providers worldwide [4]. The COVID-19 pandemic has led to complex and unpredictable conditions in every country’s health system [5]. The disease symptoms are changing, and their severity has increased periodically, adding to the challenges faced by healthcare providers, who are striving to protect their own health and the health of the community [6, 7].

During a pandemic, healthcare professionals, especially nurses, are at the forefront of patient care [8]. Nurses are the health team’s frontline professionals who are in direct contact with the community members and play a crucial role in treating and preventing the upward trend of the disease [9]. In such situations, they have many important tasks and functions such as clinical care, counseling, follow-up of the correct implementation of treatment, patient training, and teaching disease prevention methods [10]. Hence, nurses can play an essential role in controlling and preventing the virus and its complications [11].

According to the studies, it is of particular importance to consider the nursing staff’s experiences in providing care to design operational plans to combat COVID-19 [12]. Nursing is a stressful job, and those who work in this profession typically experience high levels of work stress, especially during the COVID-19 pandemic. With the special circumstances imposed by the pandemic, physical and mental distress has become very severe [8, 13]. Acute and critical conditions following the rapid spread of COVID-19, prolonged pandemic time, high rate of infectivity, lack of care equipment, especially at the beginning of the pandemic, increased number of patients, higher workload, increased number of deaths, changes in the behavior of the virus, as well as the re-infection of patients at later stages are some of the factors which could lead to serious psychological distress, including stress, anxiety, and depressive symptoms, in nurses [14, 15]. Also, the health status of the nurses of the COVID-19 wards is at risk due to the nature of their work, the use of respirators, and the risk of contamination and contaminating others, which could lead to psychological disorders [15]. These factors could cause the nurses to feel that high-risk and emergency situations would never end; these feelings can have a negative effect on the emotions of the nurses and cause burnout, boredom, and frustration, leading to feelings of helplessness and hopelessness, decreased productivity, and lower work speed [16, 17].

According to the literature, there is not enough data about nurses’ experiences in caring for patients with COVID-19 in Iran. Hence, qualitative research may provide important insights into the nurses’ experiences in taking care of COVID-19 patients.

## Methods

### Study design

A qualitative descriptive design was adopted in this study to identify nurses’ experiences in caring for COVID-19 patients. The qualitative descriptive study is concerned with understanding the individual human experience in its unique context [18]. In other words, this design is suitable for exploring nurses’ experiences of caring for COVID-19 patients in their own social context.

### Participants and setting

We selected nurses who were able to answer the research questions well enough to enhance our understanding of the phenomenon under study. Using purposive sampling method, this study was conducted on 20 nurses taking care of COVID-19 patients admitted to public hospitals affiliated with Tabriz University of Medical Sciences, Iran. The inclusion criteria were having at least 1 year work experience as a clinical nurse, experience in providing care to COVID-19 patients for at least 2 weeks, and willingness to share experiences with the researcher. Recruitment of the participants was done through professional connections, by holding a meeting with nurses who met the inclusion criteria, and by sending invitations and emails in which the objectives of the study had been described. Candidates were contacted several days later to enquire if they would like to participate in the study. A consent form was obtained from those who voluntarily accepted to participate in the study.

Data collection continued until data saturation was achieved and no new data was obtained (Interview 17). Three more interviews were conducted to confirm data saturation, but in the last three interviews, data analysis led to the emergence of repetitive codes, and no new code was obtained. The research team and two other experts reviewed the codes to confirm data saturation.

### Data collection

Since face-to-face interviews were risky during the pandemic for the researcher and the nurses, data were gathered using semi-structured and in-depth telephone interviews with the 20 eligible nurses. All the participants were informed of the aim and design of the study. Informed consent was obtained from all participants before the interview. Ethical approval was obtained from the Ethics Committee of Tabriz University of Medical Sciences. The analysis was conducted iteratively and in parallel with the interviews such that as themes emerged, they were incorporated into the next interview plan to gather more comprehensive information. Thus, as the study progressed, interviews became semi-structured and an interview guide was used to ensure that all topics were addressed. The interviews lasted for 45–60 min (mean = 52 min). Recruitment of the participants was performed through professional connections, by holding a meeting with candidates, and by sending invitations and emails in which the objectives of the study were described. Before starting each interview, the researcher explained the interview process, including arrangements for audio-recording and transcription, and obtained written informed consent from the participant. After a few warm-up questions, the participants’ demographic information was collected, and then the interview began with a general question.

There were two main questions:

1. What concerns did you have while working with a confirmed or suspected COVID-19 patient?
2. What problems did you have in the ward or the hospital?

Also, during the interview, some probing statements were made to encourage participants to provide more information and clarify their responses. Examples include, “Could you please explain it in more detail?”, “What does [x] mean?”, and “Could you give an example?” At the end of each interview, we asked, “Do you think there’s any topic the interview did not touch?” Recruitment and interviews continued until data saturation was achieved (i.e., no new themes emerged from the analysis). All interviews were recorded using a voice recorder. In one case, the participant was reluctant to allow audio-recording; thus, written notes were taken. Data collection lasted for approximately 7 months, and the entire study was conducted from April to October 2020.

### Data analysis

Taking a conventional content analysis approach, the data was analyzed using the steps proposed by Zhang and Wildemuth [19]. First, the transcript of each interview was read several times by the researcher to gain familiarity with the data and to develop a preliminary understanding of the related concepts. Then, the data was coded into semantic units (at word, sentence, and paragraph levels). The semantic units were compressed and amalgamated while their content was maintained. Finally, using continuous comparison, evaluation, feedback, and interpretation, the codes were compiled to form the categories and subcategories. MAXQDA10.0R250412 software was used to manage the data.

### Rigor

In this study, to assure the trustworthiness of the data, criteria including credibility, dependability, transferability, and confirmability, were used as proposed by Lincoln and Guba [20]. Prolonged engagement with participants may promote the credibility of a qualitative study. The researchers were in contact with the participants in the environment for 1 year to increase rapport and enable the researcher to elicit more in-depth information from the respondents. During the study, the interview transcripts, the semantic units, and the extracted codes were presented to the participants to ensure they were consistent with the participants’ experiences. All reasonable attempts were made to ensure that the participants, as a group, had maximum diversity in terms of their experience, length of service, age, and gender. Dependability was determined through a review of data and coding by a co-worker. Two external examiners with PhD degrees in nursing, who had experience in qualitative research, were asked to review the interview transcripts, the initial coding, and the categories. Any disagreements were discussed in a meeting, and a consensus was achieved among the research team about the correct coding. Regarding transferability, the characteristics of the research population and the research process were described clearly and accurately to make it possible to follow the research path and make key decisions in the analysis. The researchers actively put aside their thoughts and assumptions about the topic, recorded and documented the research procedure accurately, and refrained from deep review of texts to ensure the confirmability of the data. This was reinforced by input from the rest of the research team.

### Findings

In this study, in-depth interviews were conducted with 20 nurses (age range: 25–49 years). Findings related to the individual and professional characteristics of the participants are presented in Table 1. Data analysis revealed four themes and eight categories. The themes were ‘duality in the form of care,’ ‘confusion and ambiguity in care planning,’ ‘workload,’ and ‘social isolation in spite of positive image’ (Table 2).

**Table 1:** Demographic characteristics of the study participants

Participant Number	Age	Gender	Level of Education	Work Experience	Marital Status	Ward	History of COVID-19 Disease
1	33	Female	Bachelor of science in Nursing	10	Married	ICU	No
2	30	Male	Master of science in Nursing	7	Married	Internal Medicine	No
3	44	Female	Bachelor of science in Nursing	21	Married	Emergency	No
4	38	Male	Bachelor of science in Nursing	15	Married	CCU	No
5	49	Male	Bachelor of science in Nursing	26	Married	ICU	No
6	45	Male	Bachelor of science in Nursing	21	Married	ICU	No
7	28	Male	Bachelor of science in Nursing	4	Married	ICU	Yes
8	30	Female	Bachelor of science in Nursing	7	Single	Emergency	No
9	32	Female	Bachelor of science in Nursing	8	Single	Internal Medicine	No
10	36	Female	Bachelor of science in Nursing	13	Married	Internal Medicine	No
11	29	Female	Bachelor of science in Nursing	6	Single	ICU	Yes
12	29	Male	Bachelor of science in Nursing	6	Married	Internal Medicine	No
13	25	Male	Bachelor of science in Nursing	2	Single	ICU	No
14	27	Female	Bachelor of science in Nursing	4	Married	ICU	No
15	35	Female	Master of science in Nursing	12	Married	ICU	Yes
16	46	Female	Bachelor of science in Nursing	23	Married	ICU	Yes
17	48	Male	Bachelor of science in Nursing	25	Married	Internal Medicine	No
18	31	Male	Bachelor of science in Nursing	7	Married	ICU	No
19	30	Female	Bachelor of science in Nursing	6	Single	ICU	No
20	42	Female	Master of science in Nursing	19	Married	ICU	No

**Table 2:** Themes and categories extracted from content analysis

Categories	Themes
Duality in the form of care	Distraction from care
	Empathy and cooperation
Confusion and ambiguity in care planning	Spread of incorrect information
	Lack of scientific information
Workload	Chang in lifestyle
	Perceived care pressure
Social isolation in spite of positive image	Supportive society
	Public avoidance

### Duality in the form of care

Nursing care for patients with COVID-19 had two spectrums of distraction from care and empathy and cooperation. On the one hand, several factors such as the unknown nature of the disease for patients and fear of getting infected distracted the nurses from care provision. On the other hand, factors such as the presence of volunteer treatment staff and nursing students and people’s appreciation of the treatment staff caused empathy and cooperation among the medical staff, especially nurses.

#### Distraction from care

Analysis of the findings of this study showed that with the prevalence of COVID-19 in Iran and hospitalization of patients in wards, many factors distracted the staff from care provision. Due to political issues and to keep the society calm, during the first days of the outbreak, the treatment staff were not notified that patients with COVID-19 had been hospitalized. According to the interviews, the growing number of patients with COVID-19, the potential threats of the disease to the health of nurses and their families, and the widespread coverage of COVID-19 in the media reduced the nurses’ resilience.

*“News in the media about coronavirus, worries about one’s health and the family, and the increase in the number of patients made us suffer from psychological stress and we were distracted from care.” (Participant No. 6).*

After the hospitalization of COVID-19 patients, due to fear of the disease, some staff members refused to provide care and treatment to these patients in the wards.

“… *most colleagues were fearful of and anxious about going to the coronavirus patients' bedside* …” (Participant No. 2)

#### Empathy and cooperation

The participants in the study stated that nurses from other wards, and even other hospitals, volunteered to attend the wards to care for the admitted patients with COVID-19.

*“Nurses in other wards called me saying that they would like to work in our ward to take care of COVID-19 patients, or even students wanted to work in the COVID-19 ward ...”* (Participant No. 3)

The nurses compassionately provided care for the patients with COVID-19 despite being aware of the poor facilities and lack of equipment (masks, gauntlets, and gloves).

“*Our masks and protective clothing are indeed non-standard, but this is not a reason to abandon patients. I go to the patients and feed them, give them their medicine on time, and I am not worried about getting infected ...”* (Participant No. 6)

### Confusion and ambiguity in care planning

The COVID-19 pandemic has killed many people around the world, and with its many mutations and despite the availability of several COVID-19 vaccines, controlling the transmission chain is very difficult. This theme had two categories including ‘the spread of incorrect information’ and ‘lack of scientific information.’

### Spread of incorrect information

The results of data analysis showed that due to the limited scientific information on COVID-19, a lot of false information was shared on social media about the treatment and care of patients with COVID-19. Some fraudulent people took advantage of this opportunity by commercializing their equipment and goods in the form of traditional medicine or so-called ‘Islamic Medicine.’

*“Once, one of my friends called me and asked 'Is it true that soda is effective in treating COVID-19?' I was shocked and upset ...”* (Participant No. 14)

### Lack of scientific information

The results of data analysis showed that due to the unknown nature of the disease, different treatment and care procedures were performed, some of which resulted in serious side effects.

“*Early in the disease, there were many problems because doctors were somewhat incapable of treating the disease, which made it difficult for us to provide care* …” (Participant No. 5)

### Workload

The workload of nurses in Iran is assumed to be very high. After the outbreak of COVID-19 in Iran, this workload increased due to the increased psychological burden in the living and working environment of nurses, as well as the high care load of COVID-19 patients. This theme had two categories: ‘change in lifestyle’ and ‘perceived care pressure.’

#### Change in lifestyle

The results of data analysis showed that the nurses preferred to self-quarantine themselves in a different environment (not their own homes) to prevent the transmission of the disease to their family members and the community. Being away from the family led to stress in family members, a drop in the education quality of their children, and fear of the persistence of this situation for a long time.

“*My son’s teacher complained about his incomplete homework...”* (Participant No. 4)

“*The family also suffered from stress and anxiety and prayed a lot for us…”* (Participant No. 15)

#### Perceived care pressure

Based on the data, the non-standard design and low usability of personal protective equipment (PPE) doubled the difficulty of the nursing profession and made it difficult for nurses to provide care.

*“The equipment they gave us was of poor quality. Meanwhile, in long-term use, we have problems with nutrition and rest. Most of the time, we get headaches, nausea, skin allergies, and heavy sweating at the end of the shift. It’s hard, and I felt like I was dying.”* (Participant No. 3)

With the spread of the disease, the number of patients admitted to the wards increased, which intensified the need for care and treatment. Patients’ physical care needs included medication, management of signs and symptoms of the disease, and the psychological care needed to address death anxiety, frustration, stress, especially in the absence of family, and the fear of isolation increased the nurses’ workload.

*“At any given time, the patient may have a drop in blood oxygen saturation, which requires very rapid and immediate measures, and such issues increase the pressure of nurses’ care.”* (Participant No. 11)

*“The patient admitted to the ward is stressed and afraid of being isolated. We need to calm them down by constantly talking and explaining things. Such nursing practices have increased the workload.”* (Participant No. 7)

### Social isolation in spite of positive image

Nurses were supported by the community and praised in the media; they were called ‘health defenders’ at the beginning of the COVID-19 outbreak. However, when the treatment staff appeared in the community, people distanced themselves from them. The two categories in this theme were ‘supportive society’ and ‘public avoidance’.

### Supportive society

The results of data analysis showed that nurses were encouraged and supported by family, friends, and the community.

“*Since the start of the COVID-19 crisis, the family and people have continued to support me. This may be due to the sanctity of the field of work and the difficulty of our work because I felt it myself.”* (Participant No. 9)

Messages of appreciation were sent to nurses in the form of letters, telephone messages, or social media in different parts of the country. Nationwide, the community members and officials erected billboards thanking nurses as health defenders.

*“I was proud of all these thank-you messages ...”* (Participant No. 1)

### Public avoidance

Our results showed that nurses faced different reactions from the people in the community.

“*They only chant slogans, but it is not practiced in the society! We go places while maintaining social distance and wearing masks and gloves; when people find out that we are nurses, they behave as if we are infected individual*s.” (Participant No. 11)

Participants also reported that some of their relatives and friends cut off communication and telephone calls because they were in contact with COVID-19 patients.

“*Unfortunately, some relatives and friends avoided communication with me. They think it is only the healthcare staff that are infected with the virus! Although the virus cannot be transmitted in taxis and public places as soon as they realize that I am a nurse, they avoid me…”* (Participant No. 6)

Some of the participants reported that they had to provide a false residence address in the patient registration system of the Ministry of Health when they were infected with the virus due to people’s improper behavior.

*“When I got infected with the virus, I did not provide my correct home address because they came from the health center to disinfect the building, stairs, and other places. I was afraid that our neighbors would react badly...”* (Participant No. 16)

## Discussion

In this study, it was found that patients with COVID-19 initially remained undetected for a variety of reasons, including the lack of diagnostic kits in all cities, lack of preparedness, and lack of the necessary infrastructures. Many countries were shocked by the rapid spread of the virus in late 2019 and early 2020 [21, 22], and they were not prepared to deal with the virus due to a lack of facilities and infrastructures [23, 24].

The nurses in this study stated that despite the risk of infection and the possibility of transmission of the disease to their family members, they were at the forefront of the fight against COVID-19, and nurses from other hospital wards and even nursing students came to the battlefield to help them. Several similar studies stated that in response to this serious and unprecedented health crisis, a large number of nurses in different countries kindly came to the frontline and took care of patients with COVID-19 [25–27].

The spread of unscientific information about COVID-19 treatment in social media, as well as the lack of scientific information about care and treatment procedures in Iran, confused nurses in the provision of care programs. In a study, Catton stated that it is important to use the latest knowledge to protect healthcare professionals and nursing staff to take care of COVID-19 patients [28].

Rahmanian et al. and Rigi et al. stated that healthcare providers should be educated on the latest scientific information, especially on infectious diseases, the associated risks, standard precautions, appropriate personal hygiene measures, and related environmental measures. This is because nurses work on the frontline of care, providing direct care to individuals infected with COVID-19 [29, 30].

Liu et al. described the nurses during the COVID-19 pandemic as follows: they still applied to join the fight, took up their responsibilities, concentrated on their duties, and showed a spirit of concord and professional dedication [31].

Due to the unknown nature of the disease, different and numerous treatments were provided to patients, which led to the incidence of some complications and drug interactions. The complexities of COVID-19 treatment and care, side effects of drugs in patients, the death of young patients, the widespread suffering of patients in dire need of oxygen despite high oxygen intake (FiO2), and the frustration of treating these patients led to a perceived care pressure in nurses.

Wearing non-standard PPE and excessive sweating in them, dehydration due to sweating in personal protective clothing, lack of a specific place to feed personnel, lack of PPE, and lack of separate dirty and clean rooms were other problems that led to increased physical pressure and the burden of care in nurses.

High and intense workload drained healthcare providers physically and emotionally [31]. The findings of our study were in line with nurses’ experiences in previous epidemics of infectious diseases, such as severe acute respiratory syndrome (SARS), Middle East respiratory syndrome (MERS), Ebola, and H1N1 influenza [26, 32, 33]. In the study by Galehdar et al., it was found that the nurses experienced different kinds of psychological distress when taking care of patients with COVID-19 [25].

In line with the findings of this study, several studies reported high levels of psychological distress among nurses during outbreaks [34, 35].

Wearing non-standard PPE exposed nurses to many injuries, such as excessive sweating, a feeling of suffocation under masks, scars, etc. [36]. This can even lead to further exhaustion, hunger, and thirst of nurses.

Self-quarantine, distance from family, children’s educational decline, family-related stress, fear of the future, PPE troubles, and losing hope of treating patients led to an increase in the psychological burden on nurses. In line with this study, the studies by Shih et al. and Mok et al. showed that considering the contagious nature of the disease, nurses are forced to live away from their families as long as they take care of COVID-19 patients. In addition, the study by Ghalandar et al. showed that the nurses reported the anxiety of being separated from their children and parents and were worried about the possibility of transmitting the disease to their family members [25, 37, 38].

Nurses, with their constant presence on the treatment frontline, considered themselves responsible for taking care of patients and reducing their suffering and made great efforts to protect the people. The Supreme Leader of Iran also called the medical staff the ‘health defenders’ and acknowledged the related mortalities as martyrs.

However, the community’s attitude towards the healthcare staff in preventing transmission of the virus was different because they identified all nurses as infected people. A study by Jingoh et al. found that nurses experienced emotional stress when they heard the label ‘dirty’ or ‘clean’ or were divided into ‘approved’ and ‘suspicious’ COVID-19 groups [39].

## Conclusion

Our findings suggested that nurses lived and worked among many paradoxes during the COVID-19 pandemic. Withdrawal from care of some nurses despite empathy and cooperation of others, the presence of volunteer support forces despite the lack of equipment, the lack of scientific information and the unreliable information online, high care load in the hospital despite inadequate facilities, the nurses’ attempts to protect their families from getting infected, the physical avoidance of people in the community despite the social support in the media meant nurses provided nursing care in stressful conditions. The results of this study could provide a clearer understanding to aid the country’s managers and healthcare policymakers in taking appropriate measures to support the nurses and improve the quality of nursing care to combat COVID-19.

### Limitations

The main limitation of this study was the risk of contamination for the interviewees and the interviewer, and the need to observe social distancing. Hence, the interviews were conducted by telephone, which may have prevented a deep understanding of the situation. However, researchers tried to make the interviews as deep and effective as possible. Although we intended to examine a wide variety of nurses’ experiences in the teaching and medical hospitals of Tabriz province, the representativeness of the respondents may decrease. In the future, the participants’ experiences in different areas should be used to increase the diversity of the participants.

## Data Availability

The datasets used or analysed in the current study are available from the corresponding author upon reasonable request.

## Abbreviations

COVID-19: Coronavirus disease 2019
WHO: World Health Organization
